# Crosstalk between Brassinosteroid and Redox Signaling Contributes to the Activation of CBF Expression during Cold Responses in Tomato

**DOI:** 10.3390/antiox10040509

**Published:** 2021-03-25

**Authors:** Pingping Fang, Yu Wang, Mengqi Wang, Feng Wang, Cheng Chi, Yanhong Zhou, Jie Zhou, Kai Shi, Xiaojian Xia, Christine Helen Foyer, Jingquan Yu

**Affiliations:** 1 Department of Horticulture, Zijingang Campus, Zhejiang University, 866 Yuhangtang Road, Hangzhou 310058, China; 11416055@zju.edu.cn (P.F.); 11216063@zju.edu.cn (Y.W.); 21816059@zju.edu.cn (M.W.); 11516011@zju.edu.cn (F.W.); 21616076@zju.edu.cn (C.C.); yanhongzhou@zju.edu.cn (Y.Z.); jie@zju.edu.cn (J.Z.); Kaishi@zju.edu.cn (K.S.); xiaojianxia@zju.edu.cn (X.X.); 2 Key Laboratory of Horticultural Plants Growth and Development, Agricultural Ministry of China, Yuhangtang Road 866, Hangzhou 310058, China; 3 School of Biosciences, College of Life and Environmental Sciences, University of Birmingham, Edgbaston B15 2TT, UK; C.H.Foyer@bham.ac.uk

**Keywords:** brassinazole-resistant 1 (BZR1), *C-REPEAT BINDING FACTOR* (*CBF*) expression, *RESPIRATORY BURST OXIDASE HOMOLOG 1* (*RBOH1*), glutathione homeostasis, cold, *Solanum lycopersicum*

## Abstract

Brassinosteroids (BRs) play a critical role in plant responses to stress. However, the interplay of BRs and reactive oxygen species signaling in cold stress responses remains unclear. Here, we demonstrate that a partial loss of function in the BR biosynthesis gene *DWARF* resulted in lower whilst overexpression of *DWARF* led to increased levels of *C-REPEAT BINDING FACTOR* (*CBF*) transcripts. Exposure to cold stress increased BR synthesis and led to an accumulation of brassinazole-resistant 1 (BZR1), a central component of BR signaling. Mutation of BZR1 compromised the cold- and BR-dependent increases in *CBFs* and *RESPIRATORY BURST OXIDASE HOMOLOG 1* *(RBOH1*) transcripts, as well as preventing hydrogen peroxide (H_2_O_2_) accumulation in the apoplast. Cold- and BR-induced BZR1 bound to the promoters of *CBF1*, *CBF3* and *RBOH1* and promoted their expression. Significantly, suppression of *RBOH1* expression compromised cold- and BR-induced accumulation of BZR1 and related increases in CBF transcripts. Moreover, *RBOH1*-dependent H_2_O_2_ production regulated BZR1 accumulation and the levels of CBF transcripts by influencing glutathione homeostasis. Taken together, these results demonstrate that crosstalk between BZR1 and reactive oxygen species mediates cold- and BR-activated CBF expression, leading to cold tolerance in tomato (*Solanum lycopersicum*).

## 1. Introduction

Low temperatures (0–1 °C) represent a major environmental constraint that impacts the growth, development and productivity of many crop species. Understanding the mechanisms that underpin cold responses is vital to further optimize the breeding and production of chilling-sensitive crops. It is well established that the C-repeat binding factor/drought-responsive element binding protein (CBF/DREB)-dependent cold response pathway plays a critical role in cold acclimation in Arapidopsis (*Arabidopsis thaliana*) [1]. Cold stress activates CBF signaling pathways with the induction of *CBF1*, *CBF2* and *CBF3* expression, followed by the expression of *COLD-RESPONSIVE* (*COR*) genes [1,2]. Mutation of *CBFs* or suppression of *CBFs* transcripts decreased cold tolerance whilst overexpression of *CBFs* enhanced tolerance to this stress [3,4,5,6]. Furthermore, the CBF cold response pathway is highly conserved among plants and even is observed in the closest algal relatives of land plants [7]. Tomato (*Solanum lycopersicum*), one of the chilling-sensitive plants, fails to acclimate to cold [8]. Recently, several lines of evidence showed that previous incubation of tomato at suboptimal temperatures (8–10 °C) enhanced seedlings’ survival at a subsequent extreme temperature such as 4 °C [9]. It was also shown that transcripts of *CBF1* and *CBF2* were induced under a temperature of 10 °C, suggesting that the cold acclimation process in tomato shares some molecular mechanisms, at least involving the CBF pathway, with the cold acclimation response of Arapdopsis [9]. However, the underpinning mechanism of the activation of the CBF pathway in chilling-sensitive plants in their cold response process remains unclear.

Several transcription factors, such as inducer of CBF expression 1/2 (ICE1/2), calmodulin-binding transcription activator 1-3 (CAMTA1-3), phytochrome-interacting factor 4/7 (PIF4/7), v-myb avian myeloblastosis viral oncogene homolog 15 (MYB15) and brassinazole-resistant 1 (BZR1), positively or negatively regulate the expression of *CBF* genes by binding to their promoter regions [1,10,11,12]. Literature evidence demonstrates the involvement of phytohormones in cold responses. Exposure to cold stress leads to the expression of a number of abscisic acid (ABA) and jasmonic acid (JA) synthesis and signaling genes, resulting in a subsequent accumulation of ABA and JA [13,14]. Consistent with these findings, JA positively regulates freezing tolerance through the ICE-CBF/DREB1 cascade, whereas ABA contributes to cold tolerance via the induction of *COR* gene expression [13,14,15]. In addition, open stomata 1 (OST1), a key Ser/Thr protein kinase in ABA signaling, can interact physically with and phosphorylates ICE1 to further activate *CBF* expression in plant cold responses [16]. Emerging evidence has implicated brassinosteroid signaling in cold responses [17,18]. Lipid droplets, which play a role in BR metabolism, can be induced by cold treatment [19,20]. In tomato, the enzymes relevant for lipid droplets participate in plant biology, such as in volatile synthesis [21]. Lesions in BR biosynthesis aggravate chilling-induced injury, whilst overexpression of the BR biosynthesis gene *DWARF* or treatment with exogenous BRs enhance chilling tolerance in tomato [22]. Genome-wide analysis of Arabidopsis revealed that around ∼6% of all cold-induced genes are repressed in the BR-deficient mutant *det2-1* (*de-etiolated 2*) [23]. BRs are perceived by the transmembrane leucine-rich repeat containing receptor-like kinase brassinosteroid insensitive 1 (BRI1). The activated receptor ultimately causes the nuclear accumulation of the dephosphorylated form of the BZR1 protein and also BRI1-EMS-suppressor 1 (BES1), which directly binds the E-box (CANNTG) and BRRE (CGTGT/CG) elements of target genes [24,25]. Furthermore, BR signaling-defective Arabidopsis mutants such as *bri-1* and *bzr1* are hypersensitive to freezing, whilst an activation of BR signaling achieved by overexpression of BRI1 in Arabidopsis was found to increase freezing tolerance with enhanced expression of *CBFs* [23]. Recently, BZR1/BES1, which are major components of BR signaling, were shown to positively regulate freezing tolerance via CBF-dependent and CBF-independent pathways in Arabidopsis [26]. However, it is unclear whether BR biosynthesis is linked to the CBF pathway in response to cold stress, as is observed with that of ABA and JA.

In addition to phytohormones, reactive oxygen species (ROS) play an integral role as signaling molecules in the regulation of numerous biological processes in plants, such as growth and development, as well as responses to biotic and abiotic stimuli [27]. The respiratory burst oxidase homologues (RBOHs) play a key role in the network of ROS production in the apoplast [28]. In tomato, overexpression of BR biosynthesis gene *DWARF* increased the levels of *RBOH1* transcripts as well as the resultant increases in H_2_O_2_ accumulation, leading to cold tolerance. In contrast, a partial loss of function of *DWARF* in the *dim* mutant decreased the expression of *RBOH1*, leading to reduced cold tolerance [22]. Evidence showed that tomato *RBOH1* could be activated directly by BZR1 [29]. ROS accumulation directly regulates cellular redox status, leading to changes in the ratio of reduced glutathione (GSH) to oxidized glutathione disulphide (GSSG). In response to redox changes, cysteines (Cys) undergo a diverse spectrum of redox post-translational modifications that modulate the functions of signaling components, such as mitogen-activated protein kinase (MAPK), protein phosphatases, and transcription factors [30,31,32]. For example, ROS-related changes in glutathione homeostasis have been shown to influence the localization and function of the non-expresser of pathogenesis-related genes 1 (NPR1) protein in response to pathogen attack [33]. Interestingly, there are five and four Cys in the BZR1 protein in Arabidopsis and tomato, respectively. Cold acclimation and BR both induced an RBOH1-dependent increase in GSH/GSSG ratio, resulting in the increased stability of several redox-sensitive enzymes, such as RuBisCo activase [34,35]. In addition, H_2_O_2_ enhances BZR1 expression by promoting interactions with key regulators involved in the auxin-signaling and light-signaling pathways [36]. However, it remains unclear whether ROS participate in the regulation of BZR1 homeostasis and in *CBF* expression during BR-induced cold response.

In the present study, we show that BR participates in the regulation of basal cold tolerance by controlling the levels of *CBF* transcripts in tomato plants. Cold-induced and BR-activated accumulation of BZR1 play a critical role in the cold response by directly activating the expression of CBFs. Importantly, BZR1 triggers an accumulation of H_2_O_2_ in the apoplast, by directly binding to the *RBOH1* promoter. The resultant production of H_2_O_2_ increases the accumulation of BZR1 and *CBF* transcripts via alterations in glutathione homeostasis.

## 2. Materials and Methods

### 2.1. Plant Materials

Wild types of tomato (*Solanum lycopersicum* L. cv. Ailsa Craig, Condine Red) and *DWARF* mutant *dwf* (partial loss of function of *DWARF*, accession LA0571) in Condine Red background were obtained from the Tomato Genetics Resource Center (University of California, Davis, CA, USA). *DWARF* overexpression line (*DWF*:OE), 35S:BZR1-3HA-overexpressing lines (*BZR1*:OE) and *RBOH1*-RNAi line selected in our laboratory were used for study [22,37,38]. For generation of *bzr1* mutants, a CRISPR/Cas9 system was generated as described earlier [38]. Target sequence (GGAAGCCATCATGGAGGGAA) was designed using the CRISPR-P web tool [39]. Transgenic tomato lines were generated by the *Agrobacterium tumefaciens*-mediated transformation. The transgenic line *bzr1* has a deletion of 10 bp (ATCATGGAGG), which was 104-bp downstream from the transcription start site. Virus-induced gene silencing (VIGS) was used to suppress the transcript of *RBOH1* in *BZR1*:OE plants, *CBFs* in Ailsa Craig. The tobacco rattle virus (TRV) VIGS construct was used and *Agrobacterium-tumefaciens*-mediated virus infection was performed as described previously [6,9]. Complementary DNA fragments were PCR-amplified using the gene-specific primers listed in Table S1.

### 2.2. Growth Conditions and Treatments

Seedlings were grown in pots (12 cm in diameter) filled with a mixture of peat and perlite at a ratio of 2:1 under a 12-h/12-h light/dark cycle at 25/20 °C (day/night) and photosynthetic photon flux density of 200 µmol m^−2^ s^−1^. White light-emitting diode (LED) lamps (Philips, Holland) were used. Five-week-old plants were used for experiments. To induce cold stress, plants were firstly treated with 8 °C for one day and then treated with 4 °C for another six days under identical light conditions. Leaf samples were harvested immediately at the end of treatment for determination of electrolyte leakage and chlorophyll fluorescence. For analysis of gene expression, accumulation of BZR1 proteins as well as glutathione content, leaf samples were collected within 24 h under 8 °C. To examine the effects of exogenous BR, plants were pre-treated with 24-epibrassinolide (EBR, 200 nM) at 24 h before cold treatment. To determine the role of the glutathione redox state in BR-induced cold tolerance, WT and *RBOH1*-RNAi plants were pretreated with 6-aminonicotinamide (6-AN; an inhibitor of the pentose phosphate pathway, which produces NADPH [40]) at 5 mM, and then treated with 200 nM EBR 6 h later. To detect the effects of exogenous H_2_O_2_ and GSH on the accumulation of BZR1 protein, plants were sprayed with H_2_O_2_ at 5 mM or GSH at 5 mM with distilled water as a control. Each plant received 15 mL of the solution.

### 2.3. Determination of Relative Electrolyte Leakage

Electrolyte leakage assays were performed by determining the changes in electric conductivity (EC) by a conductivity meter (DDS-11A, Aolong, Hangzhou, China). Briefly, 0.3 g leaf tissue was placed into 50-mL tubes containing 30 mL of deionized water and the electrical conductivity (EC) was determined (S0), and the samples were shaken for one hour at room temperature before their EC was measured, giving S1. Then, the samples were boiled for 20 min and shaken for another 20 min at room temperature and their EC was measured as S2. The electrolyte leakage was calculated as: (S1-S0)/(S2-S0).

### 2.4. Analysis of Chlorophyll Fluorescence

Chlorophyll fluorescence was measured using an Imaging-PAM Chlorophyll Fluorometer (IMAG-MAXI, Heinz Walz, Effeltrich, Germany). Tomato plants were maintained in darkness for 30 min before they were used for the determination of the maximum quantum efficiency of photosystem II (*Fv*/*Fm*). The *Fv*/*Fm* was defined as (*Fm* − *Fo*)/*Fm* [41]. *Fo* and *Fm* are the minimum and maximum fluorescence after dark adaptation and they were determined under measuring light (0.1 μmol photons m^−2^ s^−1^) and saturated pulse (>4000 μmol photons m^−2^ s^−1^, 0.8 s), respectively.

### 2.5. RNA Isolation and qRT-PCR ANALYSIS

Total RNA was extracted using the RNA prep pure Plant Kit (TIANGEN, Beijing, China) and was reverse-transcribed by using Rever-Tra Ace qPCR RT Kit with genome-DNA-removing enzyme (Toyobo, Osaka, Japan). qPCR was performed on LightCycler480 detection system (Roche, Penzberg, Germany) using SYBR SuperMix (Takara, Japan). qRT-PCR was performed at 95 °C for 3 min, followed by 40 cycles of 95 °C for 30 s, 57 °C for 20 s, and 72 °C for 30 s. Quantification of relative expression level was achieved by normalization against the transcript of *ACTIN2* and *UBI3* according to Livak and Schmittgen [42]. The PCR primers used are listed in Table S2.

### 2.6. Quantification of Endogenous Brassinosteroids

The 4th leaf from the five-week-old tomato plants at 6-leaf stage was used to analyze endogenous brassinosteroids. Samples were prepared and assayed according to Luo et al. [43]. Briefly, 0.1 g fresh weight (FW) leaves was ground into powder with liquid nitrogen, and 1.0 mL of ice-cold acetonitrile (ACN) including 0.1 ng [26-^2^H_3_]-castasterone (OlChemIm, Olomouc, Czech Republic), 0.1 ng [28-^2^H_3_]-*nor*castasterone (OlChemIm, Olomouc, Czech Republic), and 0.1 ng [26-^2^H_3_] brassinolide (0.1 ng, OlChemIm, Olomouc, Czech Republic) was added as extraction solution. After overnight extraction at 4 °C, samples were centrifuged (10,000× *g*) for 10 min at 4 °C, and the obtained supernatant was mixed with 0.3 g 2-(4-boronobenzyl) isoquinolin-2-ium-modified MCX material (MCX@BBII) and 3 mL H_2_O. After centrifugation (10,000× *g*) for 1 min at 4 °C, the resulting pellets were vigorously stirred in 5 mL of 90% acetone (*v*/*v*; 0.5% formic acid was added) for 1 min. After centrifugation (10,000× *g*) for 1 min at 4 °C, the resulting pellets were vigorously stirred in 1.2 mL of 90% (*v*/*v*) acetone with 20–50 mg CH_3_COONH_4_ for 1 min. After centrifugation (10,000× *g*) for 3 min at 4 °C, the liquid phase was evaporated to dryness under mild nitrogen stream. Afterwards, sample residue was re-dissolved in 100 µL 45% (*v*/*v*) ACN and analyzed by using LC-MS/MS system. The LC-MS/MS system was a Waters Acquity UPLC/ Xevo TQ-XS triple quadrupole mass spectrometer. Chromatographic separation was performed on Acquity BEH C18 (2.1 × 100 mm, 1.7-mm column), Mobile phases consisted of 0.1% formic acid (A) and ACN (B). The gradient program was as follows: 0–3 min at 45% of B; 3–15 min from 45% to 90% of B; 15–18 min at 90% of B. The flow rate was 0.3 mL/min at 40 °C min. The electrospray ionization (positive ionization mode) conditions were as follows: capillary voltage, 3.0 kV; desolvation temperature, 550 °C. The cone and desolvation gas flows were 200 and 1000 L/h, respectively, and were obtained using a nitrogen source. Argon was used as the collision gas. The mass spectrometer was operated in selected reaction monitoring (SRM) mode. Detailed parameters for analyzing the targeted compounds by LC-MS/MS are listed in Table S3.

### 2.7. H_2_O_2_ Quantification and Cytochemical Detection of H_2_O_2_

H_2_O_2_ was extracted from 0.3-g tomato leaf samples and measured spectrophotometrically as described by Willekens et al. [44]. In brief, leaf samples were homogenized in 3 mL ice-cold 1.0 M HClO_4_; after centrifuging at 12,000 g for 5 min at 4 °C, the pH of supernatant was adjusted to 6.0–7.0 with 4 M KOH. Then, 0.5 g activated carbon was added into the supernatant. After vortexing briefly (60 s), the mixture in tubes was centrifugated at 12,000× *g* for 5 min at 4 °C followed by filtration with 0.22-µM column filter. The resulting liquid (900 µL) was then mixed with 900 µL potassium acetate buffer (100 mM, pH 4.4) containing 1 mM 2,2′-azino-bis (3-ethylbenzthiazoline-6-sulfonic acid). The reaction was initiated by adding 0.5 U horseradish peroxidase (Sigma, St Louis MO, USA). The absorbance at 412 nm was recorded and the H_2_O_2_ content was calculated from a calibration curve prepared from known concentrations of fresh H_2_O_2_.

H_2_O_2_ was visualized at the subcellular level using CeCl_3_ for cytochemistry staining [45]. Samples were fixed, embedded, sectioned, and stained for conventional electron microscopy [46]. Sections were examined under a transmission electron microscope (JEM-1200EX; JEOL, Tokyo, Japan) at an accelerating voltage of 75 kV.

### 2.8. Glutathione Content Assay

GSH and GSSG contents were determined according to the method described previously [47]. To determine GSSG, GSH was masked by 20 μL of 2-vinylpyridine. The GSH concentration was obtained by subtracting the GSSG concentration from the total concentration.

### 2.9. Western Blotting for BZR1 Abundance

For detection of BZR1 protein, the samples harvested from *BZR1*:OE plants were ground into powder in liquid nitrogen and homogenized in extraction buffer (100 mM HEPES, pH7.5, 5 mM Na_2_-EDTA, 5 mM EGTA, 10 mM Na_3_VO_4_, 10 mM NaF, 50 mM β-glycerophosphate, 1 mM phenylmethylsulphonyl fluoride, 10% (*w*/*v*) glycerol, 7.5% (*w*/*v*) polyvinylpolypyrrolidone (PVP), and 0.2% (*w*/*v*) β-mercaptoethanol), followed by centrifugation at 13,000× *g* for 20 min. To determine the concentrations of proteins, a Bio-Rad protein assay kit was used. After adjusting the concentration to the same level, proteins were then mixed with 5× loading buffer (125 mM Tris-HCl, pH6.8, 5% (*w*/*v*) SDS, 25% (*v*/*v*) glycerol, 25% (*v*/*v*) β-mercaptoethanol, 3.13 mg bromophenol blue/5 mL buffer) and heated at 95 °C for 10 min. SDS–polyacrylamide gel 12% (*w*/*v*) electrophoresis (SDS-PAGE) was performed to resolve the protein extracts. After electrophoresis, proteins were transferred to a nitrocellulose membrane and HA was identified (Pierce, 26183, Waltham, MA, USA). After incubation with a goat anti-mouse IgG antibody (Millipore, AP124P, Germany), the complexes on the blot were visualized using SuperSignalTM WEST Pico Chemiluminescent Substrate (Thermo Fisher Scientific, 34080, Waltham, MA, USA) following the manufacturer’s instructions.

### 2.10. Yeast One-Hybrid Assays

Yeast one-hybrid (Y1H) assay was performed according to the instructions of the Matchmatch™ Gold Yeast One-Hybrid System (Clontech, Mountain View, CA, USA). The promoters of tomato *CBF1* and *CBF3* were cloned into pAbAi to create the pAbAi baits and the full-length BZR1 was subcloned into pGADT7 to create the AD prey vector (primers are listed in Table S4). pAbAi baits were firstly linearized at BbsI site before they were transformed into Y1HGold and were screened on selective synthetic dextrose medium (SD) uracil. To confirm that the plasmids had integrated correctly into the genome of Y1HGold, Colony PCR analysis were performed using Matchmaker Insert Check PCR Mix 1 (Clontech, Mountain View, CA, USA). the minimal inhibitory concentrations of Aureobasidin A (AbA) for the bait strains were determined before the AD prey vectors were transformed into the bait strain. The transformants were screened on SD/-Leu/ AbA media. All transformations and screenings were repeated three times. Autoactivation and transcription factor–protein interaction analyses were carried out according to the manufacturer’s protocol.

### 2.11. Chromatin Immunoprecipitation (ChIP)

ChIP was carried out using the EpiQuiK^TM^ Plant ChIP kit (Epigentek, P-2014, Farmingdale, NY, USA) as described in the manufacturer’s protocol. Approximately 1 g of leaf samples was collected from plants at 6-leaf stage. To analyze the binding of BZR1 to *RBOH1* and *CBFs*, samples were collected from *BZR1*:OE and WT plants treated at 8 °C for 8 h. To analyze the role of *RBOH1* in BZR1 binding ability, samples were collected from *RBOH1*-silenced or non-silenced plants with application of H_2_O or 200 nM EBR for 12 h. To analyze the role of H_2_O_2_-dependent glutathione homeostasis in BZR1 binding ability, samples were collected from *RBOH1*-silenced or non-silenced plants with application of H_2_O, 5 mM H_2_O_2_, or 5 mM GSH. The DNA fragments combined with BZR1 protein were co-immunoprecipitated with an anti-HA antibody (Pierce, 26183). Goat anti-mouse IgG antibody (Millipore, AP124P, Germany) was used as the negative control. The enriched DNA fragments were quantified by qRT-PCR using the primers listed in Table S5. The ChIP-qPCR data were analyzed according to the calculation method described by Patrik [48].

### 2.12. Statistical Analysis

The experimental design was a completely randomized block design with three replicates. All statistical analyses were performed using SPSS package (SPSS 19.0, Chicago, IL, USA). The differences between the treatment means were separated by Tukey’s test at a level of *p* < 0.05 and significant differences were indicated by different letters.

## 3. Results

### 3.1. Cold-Induced BR Positively Regulates Cold Tolerance via a CBF-Dependent Pathway

**Figure 1:**
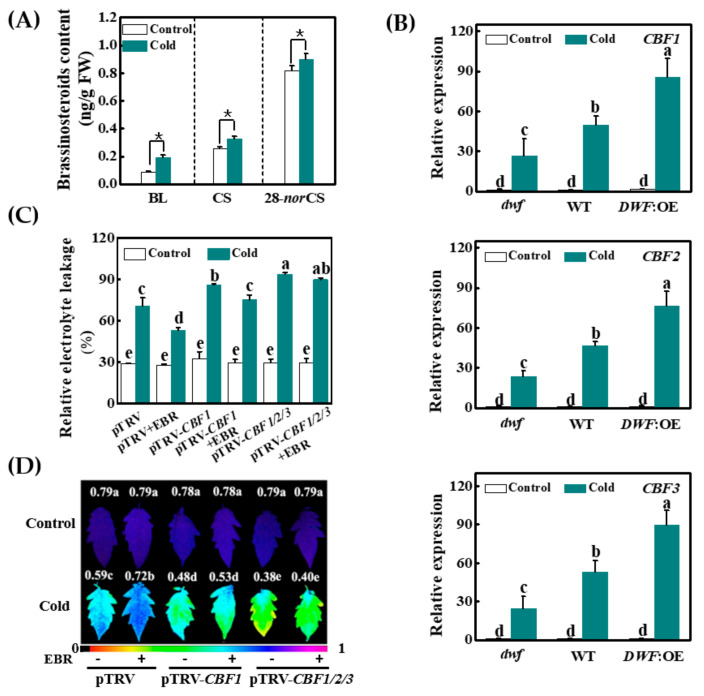
Cold-induced brassinosteroid (BR) regulated cold tolerance via c-repeat binding factor (CBF)-dependent pathway in tomato. (**A**) Cold-induced brassinosteroid accumulation. Brassinosteroid contents in tomato leaves after exposure to 25 °C (Control) and 8 °C (Cold) for 8 h. BL, brassinolide; CS, castasterone; 28-*nor*CS, 28-*nor*castasterone. * indicates significant differences according to *T*-test at 0.05% level.(**B**) The relative expression levels of *CBF* genes in *dwf* mutant, wild-type (WT), and *DWARF*-overexpressing transgenic plants (*DWF*:OE) after exposure to 25 °C (Control) or 8 °C (Cold) for 8 h. (**C**) The relative electrolyte leakage (REL) and (**D**) the maximum quantum efficiency of photosystem II (*Fv*/*Fm*) in the control (pTRV), *CBF1*-silenced (pTRV-*CBF1*), and *CBF1*/*2*/*3*-co-silenced (pTRV-*CBF1*/*2*/*3*) plants with and without exogenous BR. For cold treatment, plants were exposed to 8 °C for 24 h and subsequently exposed to 4 °C for another six days. For the exogenous BR treatment, the plants were foliar applied with 200 nM EBR 24 h before cold treatment. Plants sprayed with distilled water were used as the control. Data are the means of three biological replicates (±SD) (**A**–**C**) or eight replicates (**D**). Different letters indicate significant differences according to Tukey’s test at 0.05% level.

To determine whether plants respond to suboptimal temperatures by increasing BR biosynthesis, we analyzed leaf BR accumulation in plants with and without exposure to cold stress at 8 °C. UPLC-MS-MS analysis revealed that the content levels of brassinolide (BL), castasterone (CS), and 28-*nor*castasterone (28-*norCS*), the three detectable BRs in the leaves, were increased by 118.4%, 28.0%, and 10.2%, respectively, after exposure to cold stress for 8 h (Figure 1A, Table S6). These results suggest that the tomato plants responded to the cold acclimation temperature by increasing BR synthesis.

We then determined whether *CBFs* are involved in BR-induced cold tolerance by comparing the cold tolerance and the levels of *CBF1*, *CBF2*, and *CBF3* transcripts in the *dwf* mutant that has a partial loss of function of *DWARF*, the wild type (WT), and transgenic plants overexpressing *DWARF* (*DWF*:OE) (Figure 1B and Figure S1). Although there were no significant differences in relative electrolyte leakage (REL, indicator of membrane damage) or the maximum quantum efficiency of photosystem (PS) II (*Fv*/*Fm*, an indicator of primary photochemical energy utilization) in the three genotypes measured at 25 °C, the *dwf* mutants tended to have higher REL with lower *Fv*/*Fm* ratios after a cold stress treatment. In contrast, the *DWF*:OE plants tended to have lower REL with higher *Fv*/*Fm* ratios relative to the WT plants following exposure to cold stress (Figure S1A,B). The cold treatment significantly increased the levels of *CBF1, CBF2,* and *CBF3* transcripts in all genotypes. The induction of *CBF* expression was, however, lower in the *dwf* mutants and higher in the *DWF*:OE plants. For example, the levels of *CBF1* transcripts increased 26.5-fold in the *dwf* plants, 49.8-fold in the WT plants, and 85.7-fold in the *DWF*:OE plants, respectively (Figure 1B). Consistent with this finding, the levels of *COR47*-like transcripts, which are downstream of CBF1, were increased 2.2-fold in the *dwf* plants, 3.8-fold in the WT plants, and 8.2-fold in the *DWF*:OE plants, respectively (Figure S1C).

Next, we examined whether BR-induced cold tolerance was *CBF*-dependent by silencing *CBF1* (pTRV-*CBF1*) or co-silencing *CBF1*, *CBF2,* and *CBF3* (pTRV-*CBF1*/*2*/*3*), respectively. Transcript analysis revealed that the abundance of these target transcripts was suppressed by 70~80% in the respective silenced lines (Figure S2A). There were no significant differences in REL and *Fv*/*Fm* ratios in the empty vector plants (pTRV), pTRV-*CBF1* plants, or pTRV-*CBF1/2/3* when plants were grown at 25 °C. However, exposure to cold stress resulted in significant increases in REL and decreases in *Fv*/*Fm* ratios in the pTRV-*CBF1* and pTRV-*CBF1/2/3* plants. While exogenous application of 24-epobrassinolide (EBR, an active brassinosteroid) to leaves significantly increased the *Fv*/*Fm* ratios and decreased REL in the pTRV plants, this effect was lower in the pTRV-*CBF1* and pTRV-*CBF1/2/3* plants (Figure 1C,D and Figure S2B). Therefore, we conclude that the observed BR-induced cold tolerance is partially mediated by *CBFs* in tomato.

### 3.2. Cold and BR Increase BZR1 Accumulation

**Figure 2:**
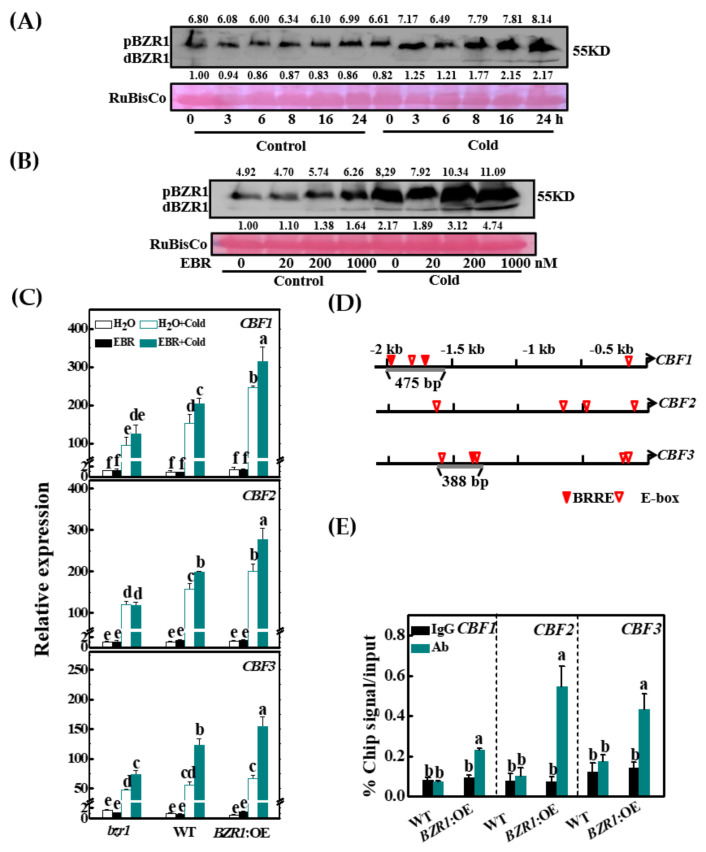
Cold-/BR-induced BZR1 functions as a transcription regulator for *CBF* expression. (**A**) The accumulation of BZR1 protein from tomato leaves exposed to 25 °C (control) or 8 °C (cold) for different periods of time. The values above and below each lane indicate the relative accumulation of pBZR1 and dBZR1, respectively. (**B**) The effect of 24-epibrassinolide (EBR) on the accumulation of BZR1 protein from tomato leaves under control and cold conditions. Plants were foliar applied with EBR at different concentrations for 24 h and subsequently exposed to cold at 8 °C for another 12 h. In addition, *35S:BZR1*-3HA-overexpressing (*BZR1*:OE) plants were used for analysis for BZR1 protein. (**C**) The transcripts of *CBFs* in *bzr1* mutant, wild-type (WT), and *BZR1*:OE transgenic plants exposed to 25 or 8 °C for 8 h; 24 h before cold treatment, the plants were foliar applied with 200 nM EBR or distilled water as the control. (**D**) BRRE and E-boxes in the tomato *CBF* promoter sequences. Numbering is from predicted transcriptional start sites. (**E**) ChIP-qPCR analysis of BZR1 binding to the promoters of *CBF1*, *CBF2,* and *CBF3* in tomato. Input chromatin was isolated from leaves exposed to 8 °C for 8 h. *CBF* promoter fragments were immunoprecipitated with an anti-HA antibody. Mouse IgG was used in control reactions. Input- and ChIP-DNA samples were quantified by qRT-PCR. The ChIP data are shown as % input DNA. Data are the means of three biological replicates (±SD) shown by vertical error bars. Different letters indicate significant differences according to Tukey’s test at 0.05% level.

We then analyzed how BR signaling was altered by the cold stress treatment. qRT-PCR analysis showed that exposure to cold did not increase the levels of transcripts encoding BZR1 (Figure S3). Under optimal temperature conditions, there were no changes in the accumulation of the phosphorylated BZR1 (pBZR1) protein over the time course of the experiment and the levels of dephosphorylated BZR1 (dBZR1) were almost below detection (Figure 2A). However, the accumulation of both pBZR1 and dBZR1 was consistently increased after exposure to the cold, especially at the 8-h time point (Figure 2A). At 25 °C, treatment with a low concentration of EBR (20 nM) did not alter the accumulation of pBZR1 or dBZR1; however, higher levels of EBR (200 and 1000 nM) increased the accumulation of both pBZR1 and dBZR1. The EBR-induced accumulation pBZR1 and dBZR1 was pronounced under cold stress (Figure 2B). Therefore, both BR and cold stimuli increased the accumulation of pBZR1 and dBZR1.

### 3.3. BZR1 Positively Regulates Cold Tolerance and the Expression of CBF Genes

Given the increased accumulation of BZR1 in response to cold stress, we then determined whether BZR1 plays a role in cold tolerance and the induction of *CBFs* using *bzr1* mutants (produced with CRISPR/Cas9), WT, and plants overexpressing *BZR1* (*BZR1*:OE). Under optimal growth temperatures, there were no significant differences in REL and *Fv*/*Fm* between the *bzr1*, *BZR1*:OE and WT plants (Figure S4). However, the cold treatment induced larger decreases in the *Fv*/*Fm* ratios and greater increases in REL in the *bzr1* mutants than in the WT plants, whereas the *BZR1*:OE plants had higher *Fv*/*Fm* ratios and a lower REL relative to the WT after exposure to cold treatment. Meanwhile, the application of EBR increased the *Fv*/*Fm* ratios and decreased REL in the WT and *BZR1*:OE plants but this effect was largely absent from the *bzr1* plants (Figure S4).

The cold-induced increases in the levels of *CBF1, CBF2,* and *CBF3* transcripts were smaller in the *bzr1* plants and greater in the *BZR1*:OE plants. For example, the levels of *CBF1* transcripts were increased 95.0-fold in the *bzr1* plants, 151.4-fold in the WT plants, and 246.8-fold in the *BZR1*:OE plants. The application of EBR to the leaves resulted in increased expression of *CBFs* in the WT and *BZR1*:OE plants but the effect was lower in the *bzr1* plants (Figure 2C). Therefore, BZR1-induced cold tolerance is associated with the activation of the CBF pathway.

### 3.4. BZR1 Directly Binds to the CBF1/3 Promoters in Tomato

We analyzed the promoter sequences of *CBFs* and found several putative BRRE and E-box motifs (Figure 2D). We ligated the 475-bp promoter fragment of *CBF1* and 388-bp promoter fragment of *CBF3* into the pAbAi vector to construct pAbAi baits. The yeast cells containing the bait vector harboring *CBF1* and *CBF3* promoter segments grew on the selection medium with 100 ng/mL AbA when transformed with BZR1-AD, while there was no growth following transformation with the empty pGADT7 vector. Therefore, BZR1 directly binds to the *CBF1* and *CBF3* promoters in vitro (Figure S5).

To confirm that tomato BZR1 binds to the promoters of *CBF1* and *CBF3*, and also *CBF2*, a ChIP-qPCR assay was performed using 35S:BZR1-3HA-overexpressing lines (*BZR1*:OE). Plants at the six-leaf stage were exposed to cold for 8 h before harvesting for analysis. The *CBF1, CBF2,* and *CBF3* promoter fragments were enriched 2.8-, 7.7-, and 8.2-fold in fractions using the anti-HA antibody in the *BZR1* overexpression lines compared to the WT plants (Figure 2E). In contrast, the IgG control antibody failed to pull down these DNA segments. Taken together, these data show that BZR1 binds to the *CBF1* and *CBF3* promoters, and potentially also the *CBF2* promoter, to activate the transcription of *CBF1, CBF2,* and *CBF3* under cold conditions, leading to cold tolerance in tomato.

### 3.5. Cold- and BR-Induced Apoplastic H_2_O_2_ Accumulation Is Dependent on the Transcriptional Activation of RBOH1 by BZR1

**Figure 3:**
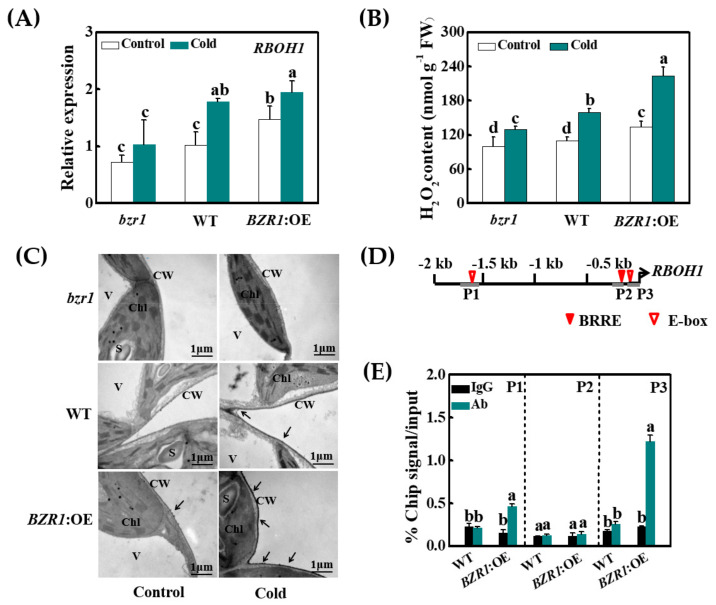
BZR1 functions as a transcription regulator for *RBOH1* expression and positively regulates apoplastic H_2_O_2_. (**A**) The relative expression level of *RBOH 1*, (**B**) accumulation of total H_2_O_2_, and (**C**) the apoplastic H_2_O_2_ in *bzr1* mutant, wild-type (WT), and *BZR1*:OE plants under control (25 °C) or cold (8 °C) conditions. Black arrows indicate H_2_O_2_ in the apoplast. Samples were collected at 8 h after cold treatment at 8 °C. V, vacuole; S, starch grain; Chl, chloroplast; CW, cell wall. (**D**) BRRE and E-boxes in the tomato *RBOH1* promoter sequence. Numbering is from predicted transcriptional start sites. (**E**) ChIP-qPCR analysis of BZR1 binding to the promoter fragments of *RBOH1* in tomato. Input chromatin was isolated from leaves exposed to 8 °C for 8 h. *RBOH1* promoter fragments were immunoprecipitated with an anti-HA antibody. Mouse IgG was used in control reactions. Input- and ChIP-DNA samples were quantified by qRT-PCR. The ChIP data are shown as % input DNA. Data are the means of three biological replicates (±SD) shown by vertical error bars. Different letters indicate significant differences according to Tukey’s test at 0.05% level.

Here, we report that the activation of BR signaling via *BZR1* overexpression enhances *RBOH1* expression and increases H_2_O_2_ accumulation in the leaf tissue and the apoplast in response to cold treatment (Figure 3A–C). In contrast, the *bzr1* mutants accumulated less H_2_O_2_ in the leaf tissue and the apoplast and they exhibited lower levels of *RBOH1* transcripts compared to the WT plants (Figure 3A–C). Given that there are several BRRE and E-box motifs in the promoter sequence of *RBOH1* (Figure 3D), ChIP-qPCR analyses were performed to verify whether BZR1 can directly bind to the *RBOH1* promoter. The P1 and P3 fragments of the *RBOH1* promoter sequences were enriched 2.3- and 4.9-fold in fractions from the *BZR1* overexpression lines compared to the WT plants using the anti-HA antibody. Furthermore, the IgG control antibody failed to pull down these DNA segments (Figure 3E). These results demonstrate that BR induces apoplastic H_2_O_2_ accumulation through direct regulation of *RBOH1* expression.

### 3.6. RBOH1 Is Required for the BZR1 Accumulation in Cold Responses

**Figure 4:**
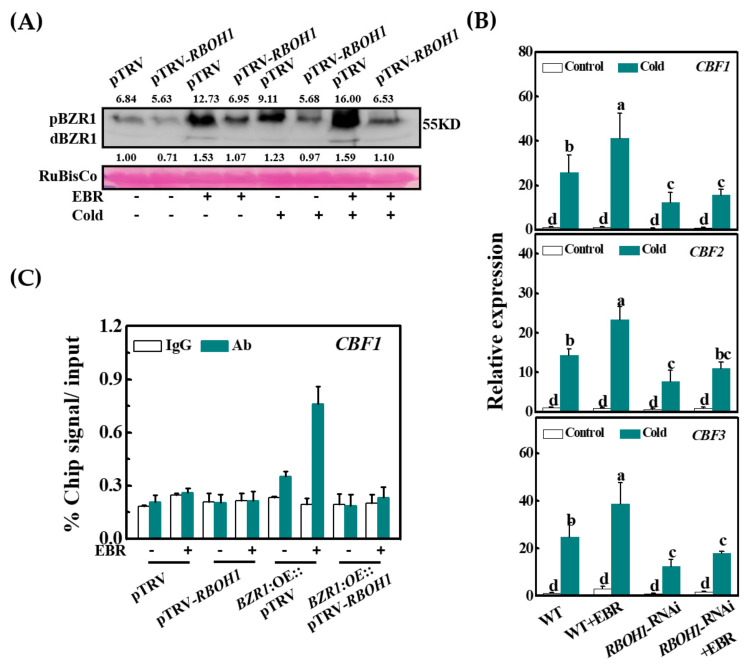
*RBOH1* plays a role in BZR1 accumulation and counteracts BR-induced *CBF* expression. (**A**) The accumulation of BZR1 protein in the control (pTRV) and *RBOH1*-silenced (pTRV-*RBOH1*) plants as influenced by the application of 24-epibrassinolide (EBR, 200 nM). Samples were collected after cold treatment at 8 °C for 12 h, and *35S:BZR1*-3HA-overexpressing (*BZR1*:OE) plants were used for VIGS. The value above and below each lane indicates the relative accumulation of pBZR1 and dBZR1, respectively. (**B**) The relative expression levels of *CBFs* in wild-type (WT) and *RBOH1*-RNAi plants. Plants were foliar applied with 200 nM EBR for 24 h and subsequently exposed to cold at 8 °C for 8 h. (**C**) ChIP-qPCR analysis showing *RBOH1* stimulation of DNA-binding activity of BZR1 in tomato. ChIP was performed with *BZR1*:OE (pTRV or pTRV-*RBOH1*) plants foliar applied with 200 nM EBR for 12 h. *CBF1* promoter fragments were immunoprecipitated with an anti-HA antibody. Mouse IgG was used in control reactions. Input- and ChIP-DNA samples were quantified by qRT-PCR. The ChIP data are shown as % input DNA. Data are the means of three biological replicates (±SD) shown by vertical error bars. Different letters indicate significant differences according to Tukey’s test at 0.05% level. VIGS, virus-induced gene silencing.

To examine whether *RBOH1* is involved in the regulation of BR signaling, we silenced the *RBOH1* gene in the *BZR1*:OE plants using VIGS, which suppressed the levels of *RBOH1* transcripts by 70% (Figure S6). Under optimal temperatures, the application of EBR to the leaves increased the accumulation of both dBZR1 and pBZR1 in the pTRV plants. However, the pTRV-*RBOH1* plants showed a lower accumulation of dBZR1 and pBZR1 than the pTRV plants in the absence or presence of EBR (Figure 4A). When pTRV plants were subjected to cold treatment for 12 h, the levels of both the dBZR1 and pBZR1 proteins were increased. However, this increase was greatest in plants pretreated with EBR. The BZR1 proteins accumulated less in *RBOH1*-silenced plants, even in the presence of the cold treatment or the EBR application (Figure 4A). *RBOH1*-dependent H_2_O_2_ production is therefore critical to the regulation of BZR1 abundance in response to both cold and BR.

We next explored whether *RBOH1* participates in the regulation of BR-induced cold tolerance and the expression of *CBF*s. Under optimal growth temperatures, there were no differences in the *Fv*/*Fm* ratios and REL between the control and *RBOH1*-RNAi plants. However, the cold treatment induced a larger decrease in the *Fv*/*Fm* ratios and a greeter increase in REL in the *RBOH1*-RNAi line compared to the WT plants (Figure S7). The application of EBR increased the *Fv*/*Fm* ratios and decreased the REL in WT plants, an effect that was much lower in the *RBOH1*-RNAi plants (Figure S7). The RNAi silencing of *RBOH1* lowered BR-induced expression of *CBF1, CBF2,* and *CBF3* (Figure 4B). The binding of BZR1 to the *CBF1* promoter was decreased by *RBOH1* silencing. The promoter fragment of *CBF1* was enriched 1.8-fold in fractions from the *BZR1*:OE plants compared with WT plants using the anti-HA antibody. However, the *CBF1* promoter fragment was not precipitated from the chromatin of the *RBOH1*-silenced plants in the absence or presence of EBR. The EBR-induced increase in the enrichment of the *CBF1* promotor fragment that was observed in fractions from the *BZR1*:OE plants was absent from the plants where *RBOH1* was silenced (Figure 4C). These results suggest that *RBOH1* participates in the regulation of BZR1 accumulation and *CBF* expression in response to cold stress.

### 3.7. BZR1 Accumulation Is Redox-Dependent

**Figure 5:**
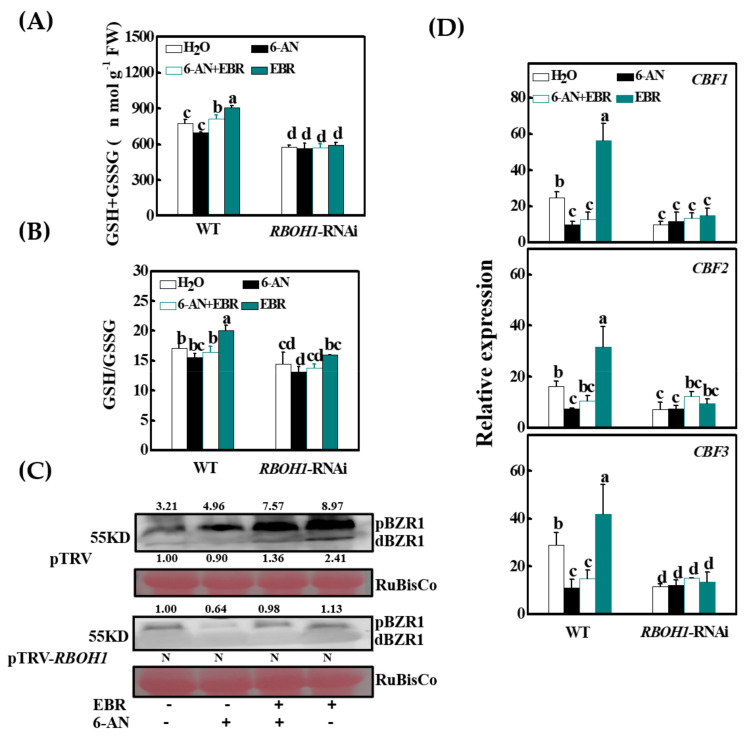
*RBOH1*-regulated BZR1 accumulation is glutathione-homeostasis-dependent. (**A**) The total glutathione contents and (**B**) molar ratio of GSH/GSSG in wild-type (WT) and *RBOH1*-RNAi plants exposed to 8 °C for 12 h. (**C**) BR enhanced BZR1 abundance by the glutathione redox status regulation, and *35S:BZR1*-3HA-overexpressing (*BZR1*:OE) plants were used for VIGS. The control plants (pTRV) as well as the *RBOH1*-silenced plants (pTRV-*RBOH1*) were foliar applied, respectively, with distilled water, 5 mM 6-aminonicotinamide (6-AN), 200 nM 24-epibrassinolide (EBR), or 5 mM 6-AN followed with 200 nM EBR for 24 h, and then subsequently exposed to cold at 8 °C for another 12 h. The value above and below each lane indicates the relative accumulation of pBZR1 and dBZR1, respectively. N means none detected. (**D**) The relative expression of *CBFs* in WT and *RBOH1*-RNAi plants exposed to 8 °C for 8 h. Data are the means of three biological replicates (±SD) shown by vertical error bars. Different letters indicate significant differences according to Tukey’s test at 0.05% level. VIGS, virus-induced gene silencing.

To determine whether *RBOH1*-mediated BZR1 accumulation is regulated by redox homeostasis, we analyzed the effects of glutathione redox homeostasis on BR-induced cold tolerance and BZR1 abundance, using 6-aminonicotinamide (6-AN, an inhibitor of the pentose phosphate pathway, which produces NADPH [40]). EBR treatment induced increases in the accumulation of total glutathione and in GSH/GSSG ratios under cold conditions, while a pretreatment of 6-AN abolished the EBR-induced increases in both parameters in the WT plants (Figure 5A,B). EBR- and 6-AN-induced changes in glutathione homeostasis were largely attributable to changes in GSH levels rather than GSSG (Figure S8). Neither EBR nor 6-AN altered the accumulation of total glutathione or the GSH/GSSG ratios in the *RBOH1*-silenced plants (Figure 5A,B). These different responses in glutathione homeostasis to EBR and 6-AN were associated with different responses of BZR1 accumulation in the WT and *RBOH1*-silenced plants. As shown in Figure 5C, pretreatment with 6-AN decreased the accumulation of the BZR1 protein in response to EBR under cold conditions in the WT plants, but these treatments had limited effects in the *RBOH1*-silenced plants. The 6-AN treatment attenuated the cold-dependent increase in *CBF1*, *CBF2,* and *CBF3* transcripts in the absence or presence of the EBR treatment in the WT plants. This treatment did not alter the levels of *CBF1*, *CBF2,* and *CBF3* transcripts in the *RBOH1*-silenced plants (Figure 5D). Similarly, EBR-induced cold tolerance was abolished by the 6-AN pretreatment in the WT plants, as indicated by the changes in REL and the *Fv*/*Fm* ratios. However, both EBR and 6-AN had little effect on REL or the *Fv*/*Fm* ratios in the *RBOH1*-silenced plants (Figure S9).

**Figure 6:**
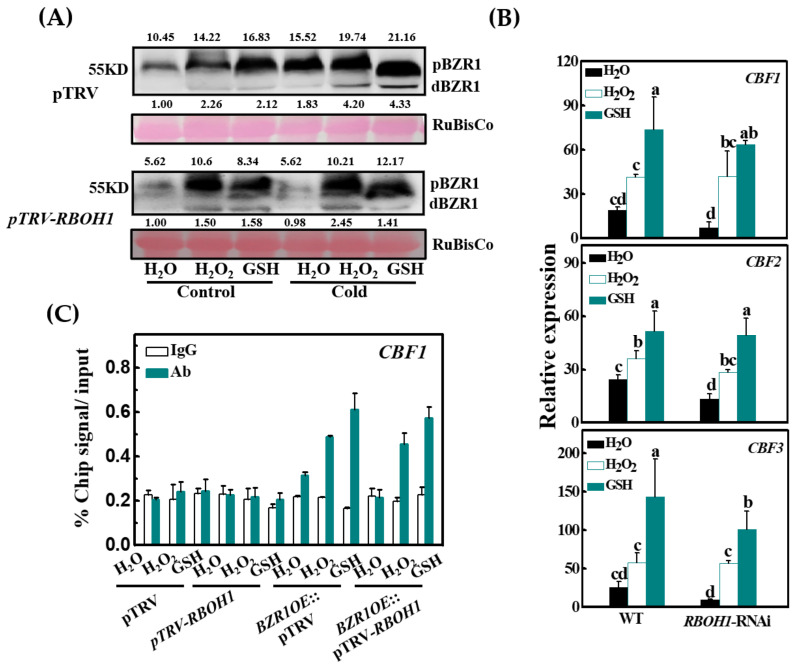
H_2_O_2_ and GSH treatment rescue the accumulation of BZR1 protein and the transcript levels of *CBF* genes in *RBOH1*-silenced plants. (**A**) The accumulation of BZR1 proteins in pTRV and pTRV-*RBOH1* plants under control (25 °C) and cold (8 °C) conditions. Plants were foliar applied with distilled water, 5 mM H_2_O_2_, and 5 mM GSH, respectively, for 12 h and subsequently exposed to 8 °C for another 12 h, and *35S:BZR1*-3HA-overexpressing (*BZR1*:OE) plants were used for VIGS. The value above and below each lane indicates the relative accumulation of pBZR1 and dBZR1, respectively. (**B**) The relative expression levels of *CBF* genes in wild-type (WT) and *RBOH1*-RNAi plants after exposure to cold at 8 °C for 8 h. Moreover, 12 h before cold treatment, plants were foliar applied with distilled water, 5 mM H_2_O_2_, or 5 mM GSH, respectively. (**C**) ChIP-qPCR analysis showing DNA-binding activity of BZR1 enhanced by H_2_O_2_ and GSH treatment in tomato leaves. ChIP was performed with *BZR1*:OE (pTRV and pTRV-*RBOH1*) plants foliar applied with H_2_O, 5 mM H_2_O_2_, or 5 mM GSH for 12 h. *CBF1* promoter fragments were immunoprecipitated with an anti-HA antibody. Mouse IgG was used in control reactions. Input- and ChIP-DNA samples were quantified by qRT-PCR. The ChIP data are shown as % input DNA. Data are the means of three biological replicates (±SD) shown by vertical error bars. Different letters indicate significant differences according to Tukey’s test at 0.05% level. VIGS, virus-induced gene silencing.

To confirm the importance of H_2_O_2_-dependent glutathione synthesis in BZR1 accumulation, we analyzed the effects of exogenous H_2_O_2_ and GSH applications on the accumulation of BZR1. As shown in Figure 6A, the application of 5 mM H_2_O_2_ or 5 mM GSH increased the accumulation of the BZR1 protein under both temperature conditions. Notably, there was a marked increase in the accumulation of the BZR1 protein in the *RBOH1*-silenced plants after treatment with either H_2_O_2_ or GSH (Figure 6A). The application of H_2_O_2_ and GSH not only increased the tolerance to cold stress in the WT plants, but also restored cold tolerance in the *RBOH1*-RNAi plants, as indicated by the increases in the *Fv*/*Fm* ratios and the decrease in REL (Figure S10). The addition of H_2_O_2_ or GSH also significantly increased the levels of *CBF1*, *CBF2,* and *CBF3* transcripts in the WT plants and in the *RBOH1*-silenced plants (Figure 6B). To validate the possible regulation of *CBFs* by H_2_O_2_-dependent glutathione homeostasis, we performed ChIP-qPCR assays to analyze BZR1 protein binding to the promoter of *CBF1* in response to the H_2_O_2_ and GSH treatments. As shown in Figure 6C, the fragment of the *CBF1* promoter was precipitated from the chromatin of both the *BZR1*:OE and *BZR1*:OE::pTRV-*RBOH1* plants using an anti-HA antibody after either H_2_O_2_ or GSH treatment, but this was not observed in the pTRV and pTRV-*RBOH1* plants (Figure 6C). Furthermore, the IgG control antibody failed to precipitate these gene promoter fragments (Figure 6C). These results show that H_2_O_2_-dependent glutathione homeostasis plays a role in the regulation of BZR1 accumulation, as well as CBF expression and cold tolerance.

## 4. Discussion

The role of BR in plant growth and development is well established and the underpinning signaling pathways have been elucidated. Although many studies have shown the positive roles of BR in plant stress responses, the molecular mechanisms that underpin BR-induced cold tolerance have yet to be established. In this study, we have shown that BR positively regulates cold responses by the BZR1-dependent activation of *RBOH1* and *CBF* expression in tomato. In addition, the data presented here show that *RBOH1* participates in the regulation of *CBF* expression and cold responses by altering the cold- and BR-induced accumulation of BZR1 involving a redox-dependent system.

In agreement with the previously reported association between BR levels and cold tolerance, the analysis of *dwf* plants reported here shows that cold tolerance was decreased in the mutants while it was increased in the *DWF*:OE plants. These findings show that the basal levels of BRs are critical to plant cold responses. Plants usually accumulate phytohormones such as ABA and JA to improve their tolerance to cold stress [23]. Previously, we found that tomato plants accumulate BR under a chilling temperature of 4 °C. We show here that a suboptimal temperature of 8 °C also induces an increase in the accumulation of BL, CS, and 28-*nor*CS (Figure 1A). These results strongly suggest that chilling-sensitive plants respond to cold also by increasing BR synthesis.

The induction of the CBF signaling pathway is one of the most important strategies employed by plants to cope with cold stress [49]. Cold stimuli rapidly induce the expression of the *CBF* genes, followed by the expression of the CBF-targeted genes in Arabidopsis, tomato, and other plant species [2,6,50]. The data presented here provide evidence for the important role of BR signaling in cold stress tolerance and in the expression of *CBFs* (Figure 1 and Figure 2, Figures S1–S4). Firstly, BR levels were positively correlated with cold tolerance and with increases in *CBF1, CBF2,* and *CBF3* transcripts. Secondly, the *bzr1* mutants had lower levels of *CBF* transcripts with decreased cold tolerance. Conversely, *BZR1* overexpression resulted in increased expression of *CBFs* together with increased cold tolerance. Thirdly, BR failed to induce CBFs in the *bzr1* mutants or cold tolerance in the *CBF*-silenced plants. These findings demonstrate that BR synthesis and signaling are important in the modulation of CBF signaling in response to cold stress. These results also demonstrate that BR regulates the expression of *CBFs* and cold tolerance in a BZR1-dependent manner.

Under optimal temperature conditions, pBZR1 is dephosphorylated to dBZR1 in response to BR, thus activating BR-directed signaling responses [51]. In contrast to these changes in the phosphorylation state of the BZR1 protein under optimal temperature conditions, there was a significant decrease in the levels of *BZR1* transcripts and a global increase in the accumulation of the pBZR1 and dBZR1 proteins in response to cold conditions (Figure 2A, Figure S3). In addition, the application of EBR further increased the accumulation of these proteins (Figure 2B). The exposure to cold did not increase the abundance of *BZR1* transcripts but rather increased the accumulation of the pBZR1 and dBZR1 proteins, especially in the presence of BR. The cold-induced and BR-promoted accumulation of these proteins contributes to the expression of *CBFs* in response to cold. While the increases in *CBF* transcripts agree well with the observed increases in BZR1 protein accumulation, both in vivo and in vitro assays confirm that the promoters of *CBF1* and *CBF3* genes are the direct targets of BZR1 (Figure 2C,D, Figure S5). Therefore, the increased accumulation of the dBZR1 protein contributes to the transcriptional activation of *CBFs* in response to cold and to BR.

*RBOH*-dependent ROS production is critical for BR-induced stress responses [46,52]. While BR induces the expression of *RBOHs*, little is known about the regulation of this process. Here, we show that the *RBOH1* gene, which is the main *RBOH* form in tomato, is the direct target of BZR1 (Figure 2D,E). Consistent with the observed changes in BZR1 levels and the abundance of *RBOH1* transcript, data are presented showing that BZR1 binds to the *RBOH1*, promoter allowing expression (Figure 2C–E). In agreement with our earlier observations, the silencing of *RBOH1* resulted in reduced tolerance to cold stress and abolished BR-induced cold tolerance [22]. Importantly, the silencing of *RBOH1* greatly decreased the accumulation of the BZR1 protein and abolished the cold- and EBR-induced expression of *CBFs.* In contrast, the application of H_2_O_2_ rescued BZR1 protein accumulation in the *RBOH1*-silenced plants under both temperature regimes (Figure 4 and Figure 6A). Taken together, these results show that crosstalk between BZR1 and H_2_O_2_ regulates the expression of *CBFs,* while BZR1-activated and *RBOH1*-dependent production of H_2_O_2_ is critical in preventing BZR1 degradation.

ROS regulate signaling proteins through modifications of protein Cys residues [31,32]. H_2_O_2_ causes oxidation of Cys residues on BZR1, thus enhancing transcriptional activity [36]. The data presented here show that H_2_O_2_ accumulation in the apoplast is essential for increasing the BZR1 protein (Figure 4A). Therefore, H_2_O_2_ has a dual role in the regulation of BZR1 at the levels of protein accumulation and also activation of protein activity. In addition to apoplastic H_2_O_2_, another secondary messanger named cyclic guanosine monophosphate (cGMP), which functions downstream of the H_2_O_2_ signal in adventitious root development, was reported to be involved in BR signaling [53]. The cGMP level at the root tip in Arabidopsis was shown to be induced by BR application and BR receptor BRI1 was found to be phosphorylated in response to cGMP [54,55]. Considering that the rapid change in the phosphorylation status of the affected proteins caused by cGMP was prevalent in the abiotic stress response, it is interesting to explore whether and how cGMP participates in the crosstalk between ROS and BR signals under cold stress.

The redox regulation of many proteins is achieved through thiol group modification [33,56,57]. Moreover, glutathionylation of thioredoxins is a redox signaling mechanism in plants [58]. The classic example of this type of regulation is the thioredoxin (TRX)-dependent activation and deactivation of Calvin–Benson cycle enzymes as well as those of the oxidative pentose–phosphate cycle [59,60]. In agreement with earlier findings showing that BR-induced increases in GSH/GSSG ratios are important for the accumulation of such redox-sensitive enzymes as RuBisCo activase, the data presented here show that *RBOH1* plays a role in regulation of BZR1 accumulation via changes in glutathione homeostasis (Figure 5 and Figure 6). Firstly, the *RBOH1*-RNAi plants had lower GSH/GSSG ratios, together with a lower level of BZR1 transcript and protein accumulation and higher expression of *CBFs* relative to the WT. Secondly, the application of exogenous BR induced an increase in the GSH/GSSG ratios, together with increased BZR1 accumulation and enhanced binding to the promoters of *CBFs.* This is not the case in the *RBOH1*-RNAi plants. Thirdly, application of 6-AN inhibited BR-induced increases in the GSH/GSSG ratios and compromised the EBR-induced accumulation of BZR1 and expression of *CBFs*. Finally, both H_2_O_2_ and GSH increased the accumulation of BZR1, enhancing the binding ability to the promoters of *CBFs* and expression of *CBFs* in the WT and *RBOH1*-RNAi plants. Given the roles of ROS and glutathione in plant growth, development, and stress responses [61], it will be of great interest to study whether this type of redox signaling also participates in the regulation of plant growth, development, and various environmental stimuli by altering the BZR1 signaling.

Notwithstanding, the current data show that BRs positively regulate tomato cold tolerance by activating the expression of *CBFs* in a redox signal-dependent pathway, in which BR central signal component BZR1 plays an important role. The detailed mechanism of the crosstalk among apoplastic H_2_O_2_, glutathione homeostasis, and BZR1 remains to be explored.

## 5. Conclusions

**Figure 7:**
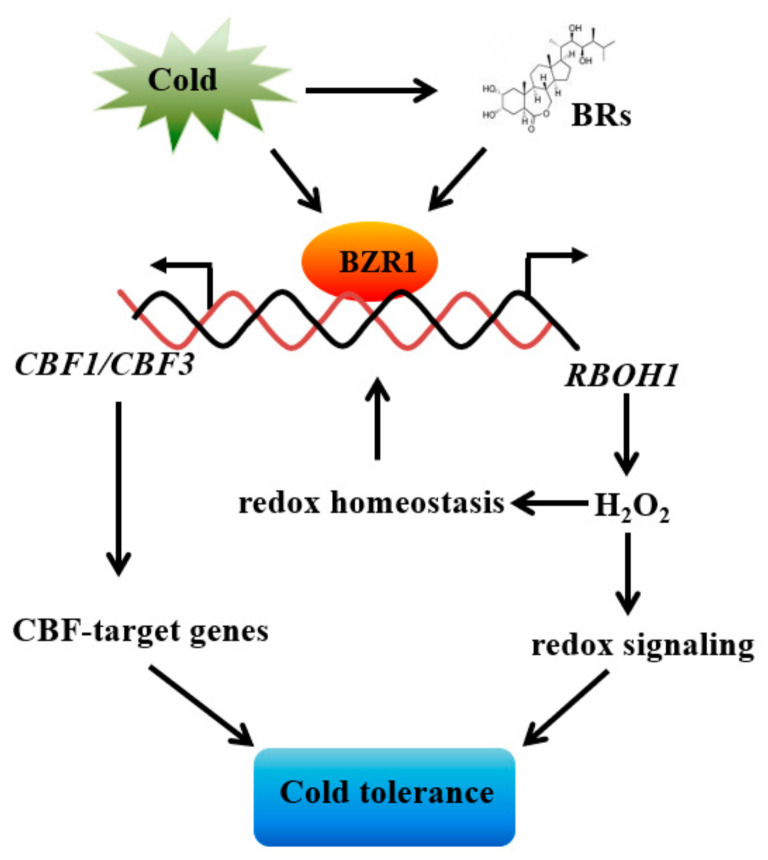
A proposed model of the crosstalk of BZR1 and ROS in *CBF* induction. Cold increases BR biosynthesis and the abundance of BZR1. While BZR1 positively upregulates the expression of *CBFs* and *RBOH1* by binding to the conserved E-box/BRRE motifs of their promoters, *RBOH1*-dependent production of apoplastic H_2_O_2_ in turn modulates the redox status to increase the BZR1 abundance, transcription of *CBF* genes, and subsequent cold tolerance.

In conclusion, we propose a model for the BZR1-dependent induction of *CBFs* (Figure 7). In this model, exposure to cold stress increases BR synthesis and the abundance of the BZR1 protein. The accumulated BZR1 protein positively regulates the expression of *CBFs* and *RBOH1* by binding to the conserved E-box/BRRE motifs of their promoters. The resultant *RBOH1*-dependent production of apoplastic H_2_O_2_ allows redox-dependent increases in BZR1 accumulation, resulting in the expression of the *CBF* genes and hence cold tolerance.

## Data Availability

All datasets generated for this study are included in the article.

## Supplementary Materials

The following are available online at https://www.mdpi.com/2076-3921/10/4/509/s1, Figure S1: Role of *DWARF* in cold tolerance in tomato, Figure S2: Silencing efficiency and the cold tolerance of *CBF1* and *CBF1*/*2*/*3* gene-silenced plants in response to EBR treatment, Figure S3: The relative expression of *BZR1* from tomato leaves exposed to 25 or 8 °C for different periods of time, Figure S4: BZR1 is essential for BR-induced cold tolerance. Figure S5: Yeast-one hybrid analysis of BZR1 binding to the promoters of *CBF1* and *CBF3* in tomato, Figure S6: Silencing efficiency of *RBOH1* in *BZR1*:OE plants, Figure S7: Role of *RBOH1* in BR-induced cold tolerance, Figure S8: The GSH and GSSG contents in WT and *RBOH1*-RNAi plants under cold condition, Figure S9: *RBOH1* plays a role in BR-induced cold tolerance by redox regulation, Figure S10: H_2_O_2_ and GSH treatment rescue the cold tolerance of *RBOH1*-silenced plants, Table S1: Primers used for VIGS constructs, Table S2: Primers used for qRT-PCR analysis, Table S3: Multiple reaction monitoring conditions used for LC-MS/MS analysis Table S4: Primers used for pAbAi-baits and AD-prey constructs, Table S5: Primers used for ChIP-qPCR analysis, Table S6: BR contents in tomato leaves under control and cold conditions.
