# Supplementary material for: Crosstalk between Brassinosteroid and Redox Signaling Contributes to the Activation of CBF Expression during Cold Responses in Tomato
Source: Antioxidants (Basel). 2021 Mar 25;10(4):509. doi: 10.3390/antiox10040509 (PMC8064343; doi:10.3390/antiox10040509)
Supplement: Supplementary file 1 [file antioxidants-10-00509-s001.pdf]

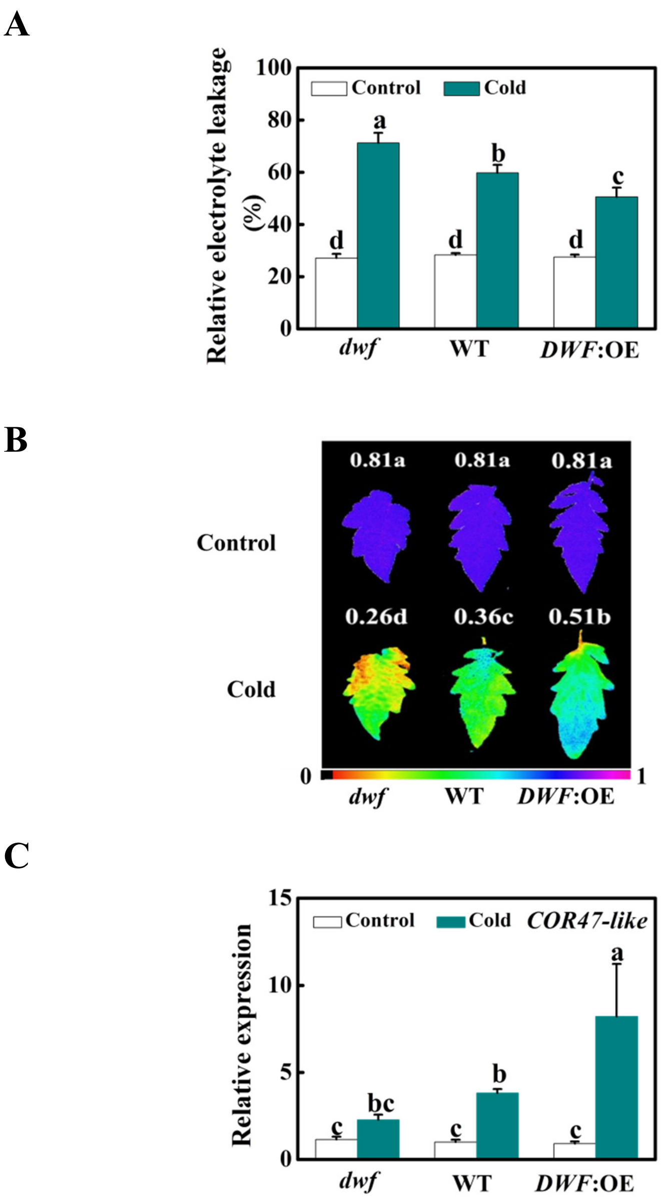

Supplemental Figure 1. Role of *DWARF* in cold tolerance in tomato.

(A-C) The relative electrolyte leakage (REL, **A**), the maximum quantum efficiency of photosystem II (*Fv/Fm*, **B**) and the relative expression level of *COR47-like* (**C**) in *dwf* mutant, wild type (WT) and *DWARF* -overexpressing transgenic plants (*DWF*: OE) with or without cold treatment. For cold treatment, plants were exposed to 8°C for 24 hours and subsequently exposed to 4°C for another six days. *COR47-like* was detected from leaf samples collected at 8 h after a cold at 8°C. Data are the means of three biological replicates (±SD) (A, C) or eight replicates (B). Different letters indicate significant differences according to Tukey's test at 0.05% level.

A

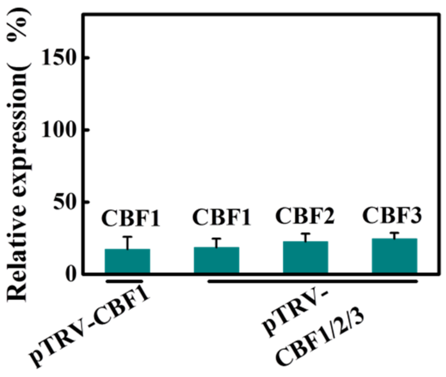

B

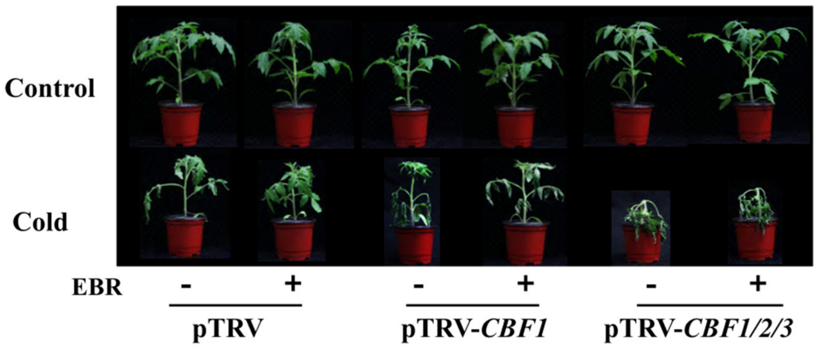

Supplemental Figure 2. Silencing efficiency and the cold tolerance of *CBF1* and *CBF1/2/3* gene silenced plants in response to EBR treatment.

(A) The relative expression levels of *CBF1* and three *CBF*s in *CBF1* silenced (pTRV-*CBF1*) and *CBF1/2/3*-cosilenced (pTRV-*CBF1/2/3*) plants respectively. Relative gene expression was calculated using the control (pTRV) plants as 1. **(B)** The phenotypes of pTRV-*CBF1* and pTRV-*CBF1/2/3* plants with or without cold treatment. For cold treatment, plants were exposed to 8°C for 24 hours and subsequently exposed to 4°C for another six days. 24 hours before cold treatment, the plants were pre-treated with 200 nM 24-epibrassinolide (EBR) or distilled water as the control.

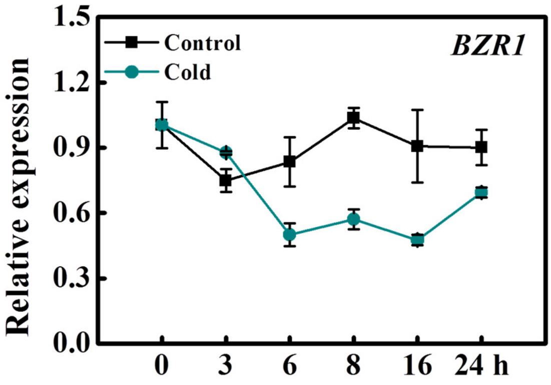

Supplemental Figure 3. The relative expression of *BZR1* from tomato leaves exposed to 25℃ or 8℃ for different periods of time.
Data are the means of three biological replicates (±SD) shown by vertical error bars.

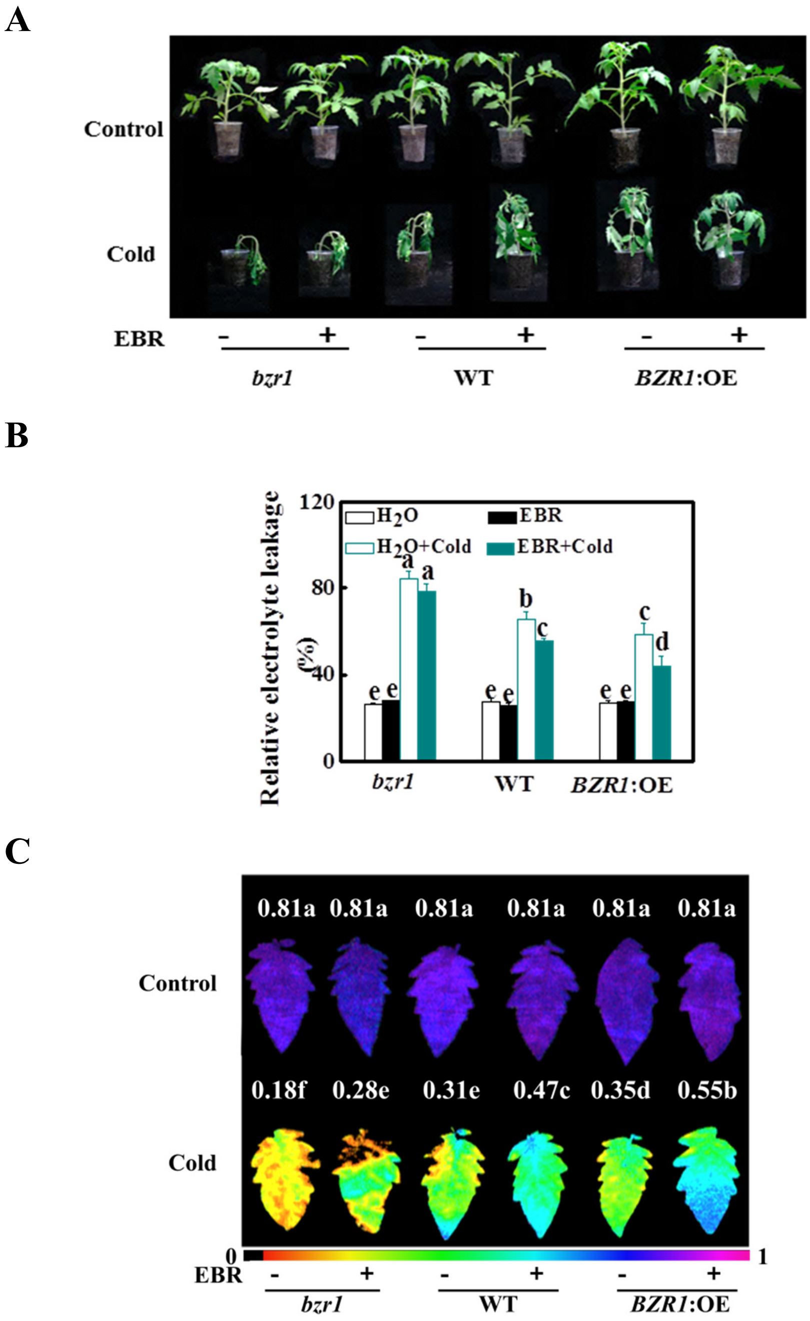

Supplemental Figure 4. *BZR1* is essential for BR-induced cold tolerance.
(A-C) The phenotypes (**A**), relative electrolyte leakage (REL, **B**) and maximum photochemical efficiency of PSII (*Fv/Fm*, **C**) in *bzr1* mutant, wild type (WT) and *35S:BZR1*-3HA-overexpression plants (*BZR1*:OE) with and without cold treatment. For cold treatment, plants were exposed to 8°C for 24 hours and subsequently exposed to 4°C for another six days. 24 hours before cold treatment, the plants were foliar applied with 200 nM 24-epibrassinolide (EBR) or distilled water as the control. Data are the means of three biological replicates (±SD) (B) or eight replicates (C). Different letters indicate significant differences according to Tukey's test at 0.05% level.

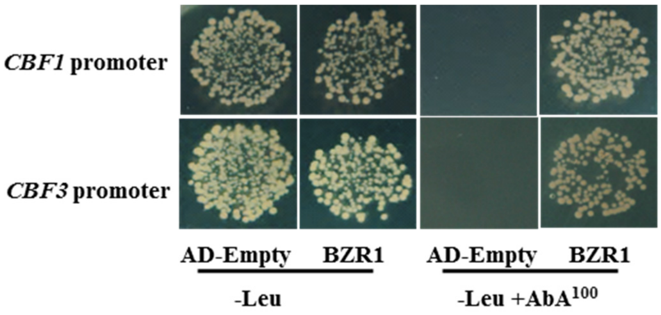

Supplemental Figure 5. Yeast-one hybrid analysis of BZR1 binding to the promoters of *CBF1* and *CBF3* in tomato.

The 475 - and 388 bp fragments from *CBF1* and *CBF3* promoter sequences indicated in (Figure 2D) were cloned into pAbAi vector respectively. Interaction was determined on SD medium lacking leucine in the presence of 100 ng/ml Aureobasidin A (–Leu+AbA^{100}). AD-empty and pAbAi-*CBFs* were used as negative controls.

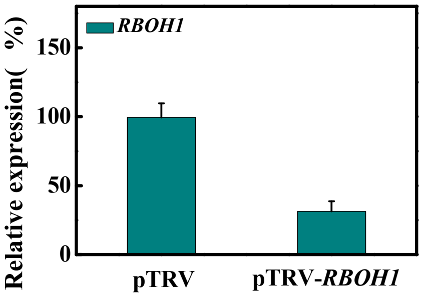

Supplemental Figure 6. Silencing efficiency of *RBOH1* in *BZR1*:OE plants.
Relative gene expression was calculated using the pTRV plants as 1.

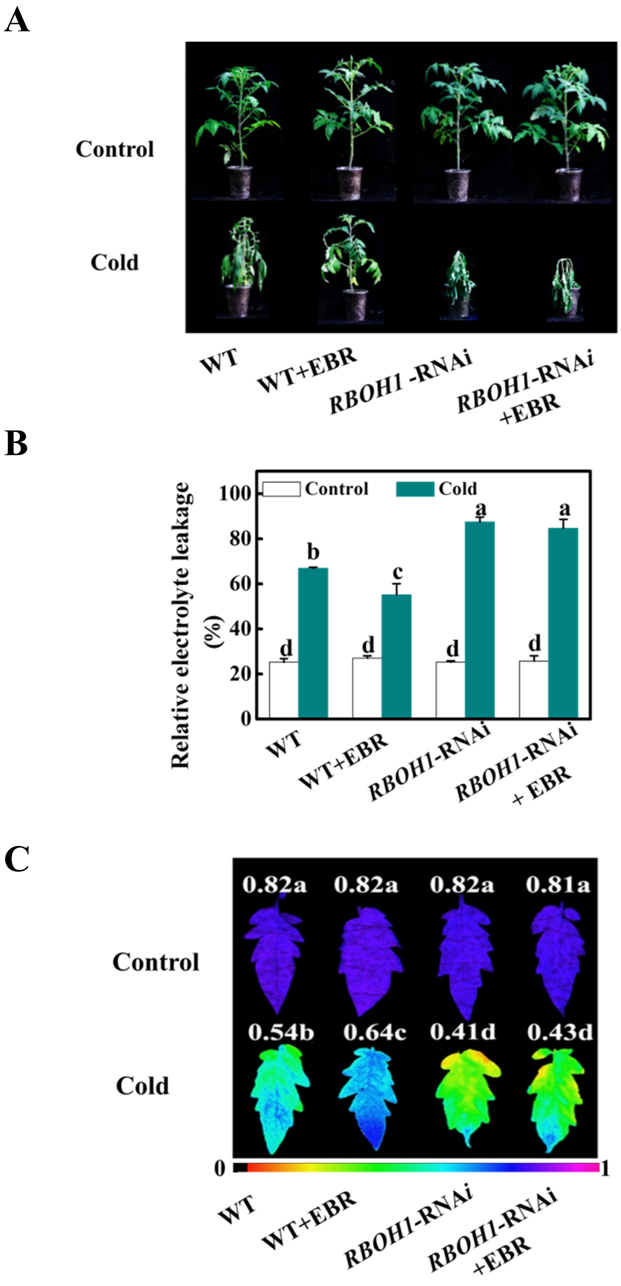

Supplemental Figure 7. Role of *RBOH1* in BR-induced cold tolerance.

(A-C) The phenotypes (**A**) relative electrolyte leakage (REL, **B**) and maximum photochemical efficiency of PSII (*Fv/Fm*, **C**) in the WT and *RBOH1*-RNAi plants with or without cold treatment. For cold treatment, plants were exposed to 8°C for 24 hours and subsequently exposed to 4°C for another six days. 24 hours before cold treatment, the plants were foliar applied with 200 nM 24-epibrassinolide (EBR) or distilled water as the control. Data are the means of three biological replicates (±SD) (B) or eight replicates (C). Different letters indicate significant differences according to Tukey's test at 0.05% level.

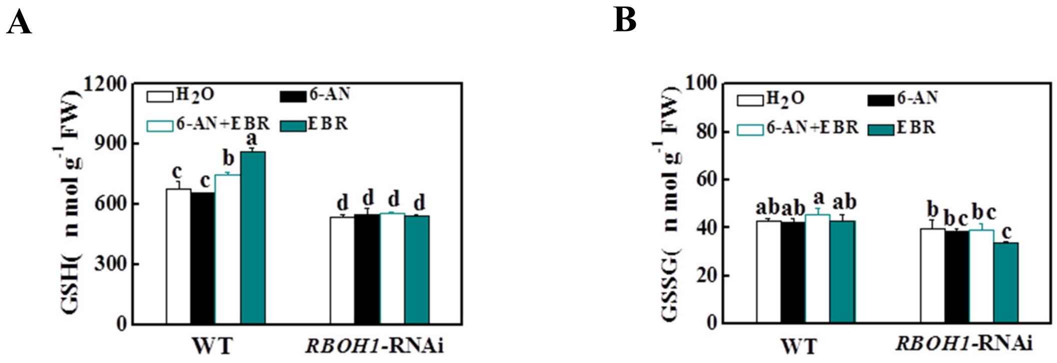

Supplemental Figure 8. The GSH and GSSG contents in WT and *RBOH1*-RNAi plants under cold condition.

(A and B) The GSH (**A**) and GSSG contents (**B**) in WT and *RBOH1*-RNAi plants exposed to 8°C for 12 hours. 24 hours before cold treatment, the plants were foliar applied with distilled water, 5 mM 6-aminonicotinamide (6-AN), 200 nM 24-epibrassinolide (EBR) or 5 mM 6-AN followed with 200 nM EBR, respectively. Data are the means of three biological replicates (±SD) shown by vertical error bars. Different letters indicate significant differences according to Tukey's test at 0.05% level.

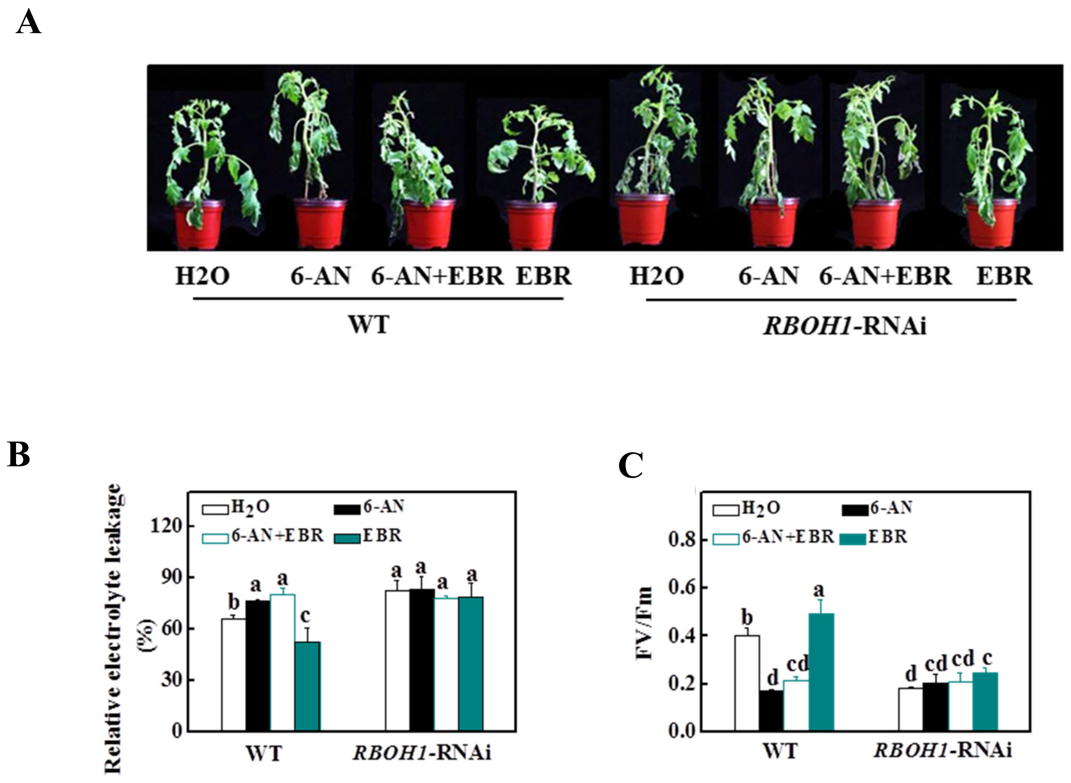

Supplemental Figure 9. ***RBOH1*** **plays a role in BR-induced cold tolerance by redox regulation.**

(A-C) The phenotypes (**A**), relative electrolyte leakage (REL, **B**) and maximum photochemical efficiency of PSII (*Fv/Fm*, **C**) in wild type (WT) and *RBOH1*-RNAi plants with and without cold treatment. For cold treatment, plants were exposed to 8°C for 24 hours and subsequently exposed to 4℃ for another six days. 24 hours before cold treatment, the plants were foliar applied with distilled water, 5 mM 6-aminonicotinamide (6-AN), 200 nM 24-epibrassinolide (EBR) or 5 mM 6-AN followed with 200 nM EBR, respectively. Data are the means of three biological replicates (±SD) (B) or eight replicates (C). Different letters indicate significant differences according to Tukey's test at 0.05% level.

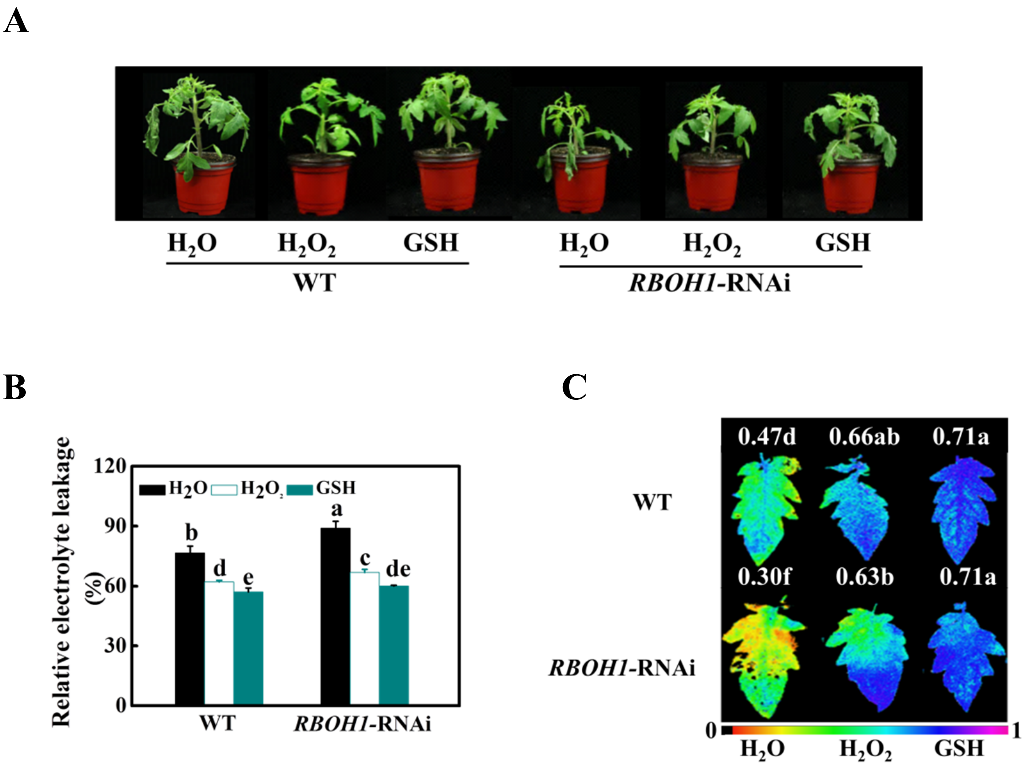

Supplemental Figure 10. H_2O_2 and GSH treatment rescue the cold tolerance of *RBOH1*-silenced plants.

(A-C) The phenotypes (**A**), relative electrolyte leakage (REL, **B**) and maximum photochemical efficiency of PSII (*Fv/Fm*, **C**) in wild type (WT) and *RBOH1*-RNAi plants with and without cold treatment. For cold treatment, plants were exposed to 8°C for one day and subsequently exposed to 4℃ for another six days. 12 hours Before cold treatment, Plants were foliar applied with distilled water, 5 mM H_2O_2 and 5mM GSH respectively. Data are the means of three biological replicates (±SD) (B) or eight replicates (C). Different letters indicate significant differences according to Tukey's test at 0.05% level.

Supplemental Table 1. Primers used for VIGS constructs

Vector	Forward primer	Reverse Primer
pTRV2-*CBF1*	5’-CCGgaattcTATGCTACCTCCACCT-3’	5’-GCtctagaAACCCAACAAGTTTCT-3’
pTRV2-*CBF1/2/3*	5’-CGCggatccAGGGGAATCAGGAAGAGGAAT-3’	5’-GCtctagaGAAGATTTCGACGGCCTGAG-3’
pTRV2-*RBOH1*	5’-CGCgagctcCGTTCAGCTCTCATTACCATGG-3’	5’-CCGctcgagCCGAAGATAGATGTGTGTACCG-3’

Supplemental Table 2. Primers used for qRT-PCR analysis

gene	Accession number	Forward primer	Reverse Primer
CBF1	Solyc03g026280	5’-GTGACTTCGTGGATGAGGAG-3’	5’-AGGCATCAGTTTCCACACAA-3’
CBF2	Solyc03g124110	5’-TTCGATCGGAAGAAGTTTCA-3’	5’-CAAGTAATCCTGGCATGGAA-3’
CBF3	Solyc03g026270	5’-TGCCGGGTTTACTTACGAAT-3’	5’-TCAGCTTCCACATGATCTCC-3’
RBOH1	Solyc08g081690	5’-TCCAGCACAAGATTACCG-3’	5’-CCTCCATTGCGACGAT-3’
BZR1	Solyc04g079980	5’-TAGCCCGATTCCATCTTACC-3’	5’-TAATGGTGGTAGCGACAAGG-3’
COR47-like	Solyc04g082200	5’-TCTAGTAGCTCCAGTGATG-3’	5’-TCTCCTCTGTTTCCTCGT-3’
ACTIN	Solyc11g005330	5’-TGTCCCTATTTACGAGGGTTATGC-3’	5’-CAGTTAAATCACGACCAGCAAGAT-3’
UBI3	Solyc01g056940	5’-GCCGACTACAACATCCAGAAGG-3’	5’-TGCAACACAGCGAGCTTAACC-3’

Supplemental Table 3. Multiple reaction monitoring conditions used for LC-MS/MS analysis

Compound	Precursor（m/z）	Ion product（m/z）	Cone (V)	Collision Energy (eV)
brassinolide	708.43	160.96	70	50
[26-$^{2}H_3$]-brassinolide	711.43	160.96	70	50
castasterone	692.5	160.96	75	45
[26-$^{2}H_3$]-castasterone	695.5	160.96	75	45
28-*nor*castasterone	678.46	160.96	50	45
[28-$^{2}H_3$]-norcastasterone	681.46	160.96	50	45

Supplemental Table 4. Primers used for pAbAi-baits and AD-prey constructs

Vector	Forward primer	Reverse Primer
pAbAi-*CBF1*	5’-GGggtaccTACACATGTTTCTCAATTTTACA-3’	5’-CCctcgagTTGAAAAGATAGTGGAAGGT-3’
pAbAi-*CBF3*	5’-GGggtaccGAGATTTTACGTGTCGTTCTGTTGA-3’	5’-CCctcgagGGCCTGATCAATTGGTTAGGATG-3’
pGADT7-*BZR1*	5’-CCcatatgATGTGGGAAGGTGGAGGGTTG-3’	5’-CGCcccgggTCACATCCGAGCAGTCCCAC-3’

Supplemental Table 5. Primers used for ChIP-qPCR analysis

DNA fragments	Forward primer	Reverse Primer
CBF1	5'-GTTTCTCAATTTTACACGTG-3'	5'-GATATGCTTGGAATTGG-3'
CBF2	5'-TAGAAAGTTTGCCACAT-3'	5'- CGGTATTACACGGAGTT-3'
CBF3	5'-GTTAGACGCACGGAAGAT-3'	5'-GAACACGGAGTTAGAGGG-3'
P1	5'-ATCCTGACTCCAACACGACT-3'	5'-GGTCACAAACTTAGCTTGAACG-3'
P2	5'-CTTTTGTTTTGCTATTGGTA-3'	5'-CGTAAAGAAAACCATAAATC-3'
P3	5'-CATCTGTTTCATTCTATACGAGTC-3'	5'-AATGGTGAGGAAGTGAGGGT-3'

Supplemental Table 6. BR contents in tomato leaves under control and cold conditions

Treatment	Brassinolide （ng/g FW）	castasterone（ng/g FW）	28-*nor*castasterone（ng/g FW）
25℃	0.087±0.008	0.257±0.011	0.816±0.033
8℃	0.191±0.018	0.325±0.017	0.900±0.041
